# Necrotizing fasciitis in a young patient with acute myeloid leukemia – a diagnostic challenge

**DOI:** 10.1186/1754-9493-8-28

**Published:** 2014-06-26

**Authors:** Mareen Brumann, Viktoria Bogner, Andreas Völkl, Karl Sotlar, Ekkehard Euler, Wolf Mutschler

**Affiliations:** 1 Department of Trauma Surgery, University Hospital Munich, Ludwig-Maximilians-University, Nussbaumstr. 20, Munich 80336, Germany; 2 Department of Hematology and Oncology, University Hospital Munich, Ludwig-Maximilians-University, Ziemsenstr. 1, Munich 80336, Germany; 3 Institute of Pathology, University Hospital Munich, Ludwig-Maximilians-University, Thalkirchnerstr. 36, Munich 80337, Germany

**Keywords:** Necrotizing fasciitis, Soft tissue infection, Acute myeloid leukemia, Granulocytopenia, Diagnostic difficulties

## Abstract

**Background:**

Necrotizing fasciitis is characterized by a fulminant destruction of the soft tissue with an alarmingly high mortality rate. One of the main reasons for the continued high mortality is due to the challenge to punctual recognize and diagnose this disease, as specific cutaneous signs can vary or even be missing early in its evolution – especially in case of simultaneous first manifestation of an acute leukemia.

**Case presentation:**

An untypical case of necrotizing fasciitis disease in a young patient with the first diagnosis of acute myeloid leukemia is presented. After her induction chemotherapy the only presenting clinical sign was fever in the presence of severe neutropenia without an evident infectious focus. After a few days a painless confluent, erythematous, pustular skin rash with a central necrosis on lateral thigh appeared. Escherichia coli was isolated from blood cultures. Surgical debridement was performed and showed subcutaneous tissue, fascia and underlying muscle around the site of initial cutaneous manifestation with typical necrosis on exploration. But, initially taken skin biopsy did not show any typical histopathological findings like bacteria or inflammatory cells confirming necrotizing fasciitis. Nevertheless, the intraoperative findings were impressive and highly indicative for a necrotizing soft tissue infection, so that the patient was treated according to clinical guidelines with extensive recurrent surgical debridement, broad-spectrum antibiotics and intensive care therapy. After recovering from NF, she successfully underwent further chemotherapy and stem cell transplantation.

**Conclusion:**

The presented case highlights the risk of potential misinterpretation, delayed diagnosis and treatment of necrotizing fasciitis in patients presenting with an untypical clinical and histopathological manifestation of necrotizing fasciitis as a result of severe neutropenia following chemotherapy for acute myeloid leukemia.

## Background

With an incidence of 0.04 per 1000 person-years, necrotizing fasciitis (NF) is a rare but fulminant and even life threatening soft tissue infection [1]. Despite increasing knowledge about its etiology, predictors, progressive clinical manifestation and improvements in diagnosis and therapy, the mortality rate still remains - subject to its stage and localization - 15 to 46% [2]. NF is characterized by a rapid onset of extending inflammation and necrosis involving fascia, muscles and subcutaneous tissues with a successive affection of the overlying skin. According to microbiological findings it is classified into two types. Type I NF is a polymicrobial infection, whereas type II NF presents as a monomicrobial bacterial infection most commonly caused by group A β-hemolytic streptococci. In most cases, the clinical findings are inadequate pain (“pain out of proportion”) regarding the minimal changes of the cutis. Therapy consists of immediate and radical surgical debridement combined with empirical broad-spectrum antibiotic coverage. Although multidisciplinary therapy protocols have led to a decrease of mortality rate in the last decades, there is an increasing number of immunosuppressed patients (17%) and patients suffering from hematological malignancies (5%) who are particularly at risk [3]. Especially, as these patients do not always present a “typical” etiopathology, NF remains a critical diagnostic and therapeutic challenge requiring an early and aggressive surgical intervention for salvage. We report a case of severe necrotizing fasciitis of the right lower extremity in a young female patient with acute myeloid leukemia.

## Case presentation

A 30-year-old woman from Saudi-Arabia was admitted to our department of internal medicine with the strong suspicion of first manifestation of acute myeloid leukemia. Laboratory testing showed mild anemia and thrombocytopenia with normal leukocyte count. The differential blood cell count, however, showed 47% leukemic blasts. A bone marrow biopsy was performed immediately. With approximately 90% blasts the diagnosis of acute myeloid leukemia (AML) was confirmed. Flow cytometry showed an expression of CD34, HLA-DR, CD38 and cyMPO. A cross-lineage of CD56 was found, which correlates with a poor prognosis [4]. Cytogenetics and molecular analysis of the bone marrow cells revealed an ETV6-gen deletion in 12p13. According to the hematologic protocol intensive induction chemotherapy with HAM (high-dose Ara-C and Mitoxantron) was immediately initiated.

**Figure 1:**
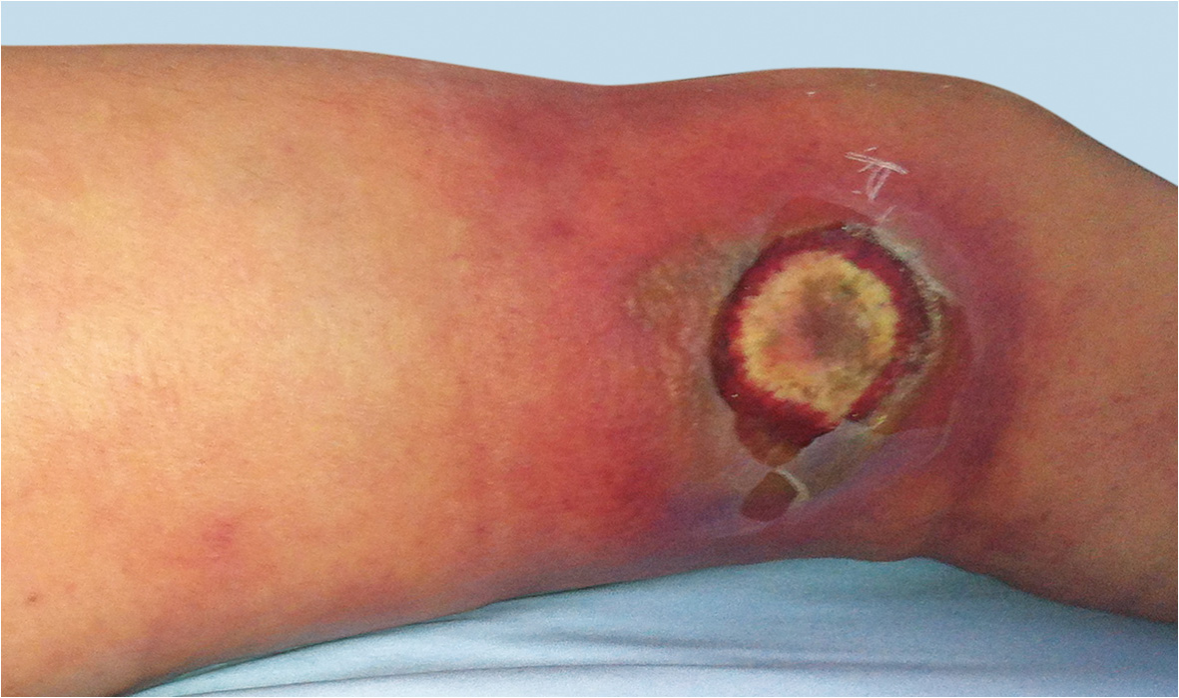
Skin manifestation after bagatelle injury on day 11 after admission.

**Figure 2:**
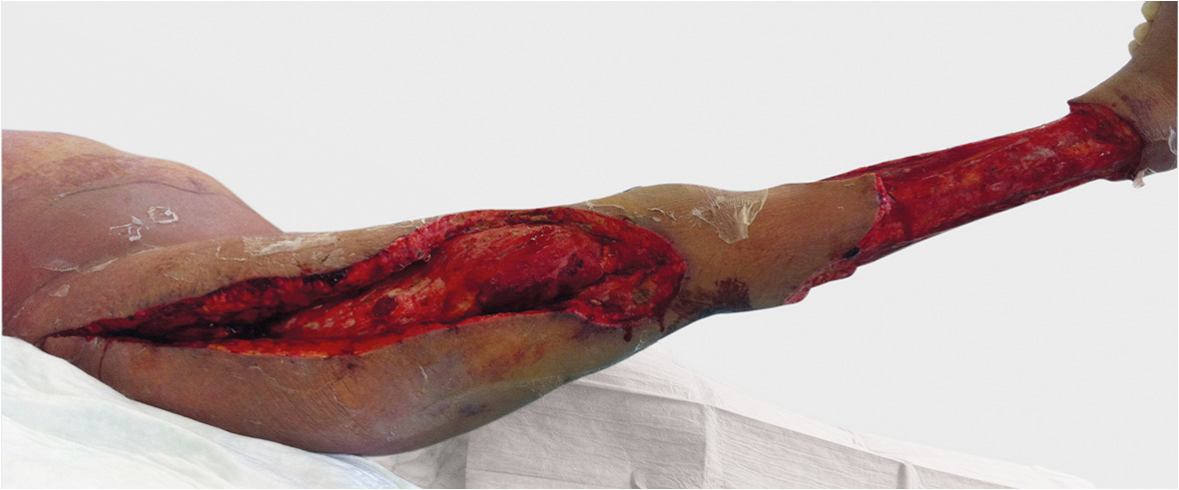
Intraoperative appearance after initial radical debridement on day 11 after admission.

**Figure 3:**
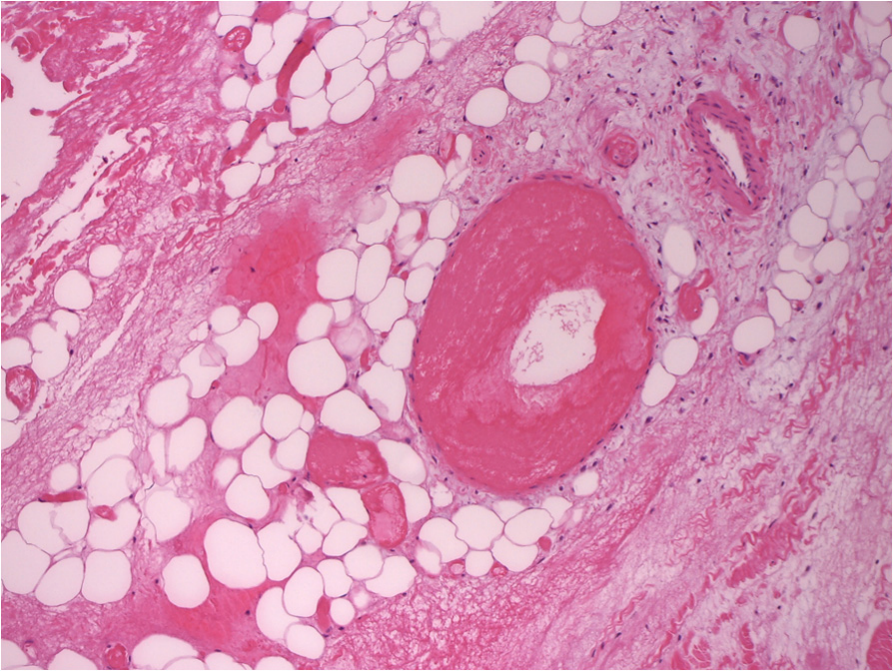
Areactive necrosis of subcutaneous fat and fascia without detection of bacteria (H&E, 200×).

**Figure 4:**
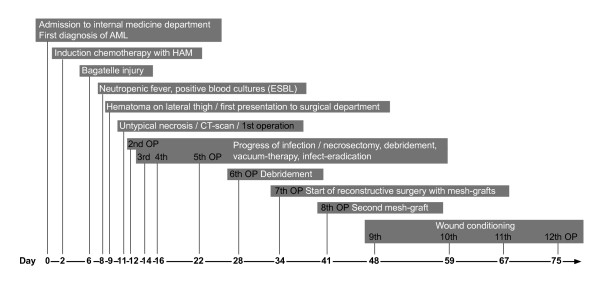
Diagram of the course of disease.

On day 8 after admission (2 days after the first cycle of chemotherapy was finished) the patient developed neutropenic fever up to a temperature of 39.4°C. The first laboratory testing showed a decrease in leukocytes down to 0.4 G/l with a C-reactive protein (CRP) less than 0.1 mg/dl. Escherichia coli with an extended-spectrum beta-lactamase (ESBL) was isolated from initially taken blood cultures, whereas all other clinical and diagnostic examinations (x-ray of the chest, urine, echocardiography and ultrasonography) were normal. Empirical broad-spectrum antibiotics was immediately initiated with Piperacillin/Tazobactam and switched to Meropenem after microbiologic testing confirmed ESBL sepsis. On that first day of fever, the patient told us, that she fell in the bathroom hurting her right knee two days before (day 6 after admission). There was no apparent injury on her leg. On further clinical examination, besides fever, her vital signs were always normal. In the course of the next 2 days neutrophils dropped below 0.1 G/l and CRP levels soared to a peak of 29.9 mg/dl. Highest procalcitonin level (PCT) measured was 1.7 ng/ml. Three days after the patient suffered from the minor injury to her lateral thigh, she suddenly presented a small hematoma with only little pain (day 9 after admission). There were no signs of superinfection of the hematoma explaining the clinical and laboratory signs of this unclear systemic infection. On that day, the patient was first presented to the surgical department. Within the next two days, the hematoma rapidly changed to a big untypical necrosis of the skin (day 11 after admission, see Figure 1). Therefore, the patient was again presented to the surgical department. The patient was still in a good general condition with only little pain. A CT scan was initiated and showed entrapped air under the site of necrosis. Although the skin manifestation was not typical for a necrotizing soft tissue lesion, we considered the possibility of an untypical case of NF during severe neutropenia. Therefore, a surgical exploration was performed immediately (see Figure 2). Macroscopically, subcutaneous tissue, fascia and muscle showed the typical characteristics of necrotizing fasciitis with grey necrotic tissue, facial edema, vessel thrombosis and non-contacting muscle. Intraoperative, there was only minimal resistance dissecting the subcutaneous fascia off the deep fascia, which is commonly described as positive “finger test” [5]. The histological findings showed facial necrosis, vasculitis, thrombosis and suppuration of the veins and arteries coursing through the fascia. But, there were no signs of disease-causing bacteria or infiltration of polymorphonuclear leukocytes in the deep dermis and fascia as expected. First microbiological testing did not find any bacteria. The initial histopathological report could not confirm the typical picture of necrotizing fasciitis (see Figure 3). In the further course of time, intensive care medicine was needed. After confirmation of adequate morphologic blast clearance in the bone marrow supportive treatment with granulocyte colony stimulating factor (G-CSF) was added on day 18 (after start of chemotherapy). Neutrophils eventually recovered on day 23 (>500/μl). After beginning to recover, histopathological analyses of additional specimen of surgical resections showed the expected typical pathological findings like inflammatory cells and bacteria. Furthermore, microbiological testing confirmed ESBL infection. A multiplicity of operations with extended debridement was performed in order to eradicate the infection using negative pressure wound therapy. After successful eradication the skin defect involved the complete lateral thigh including nearly the whole circumference of the lower leg. In the further course of time, the immense skin defects had to be prepared for further reconstructive surgery. Reconstructive surgery with mesh graft harvest was complicated by the limited residual skin available on the lower extremity and the delay on wound healing of the harvest sites. Due to unfavorable cytogenetics and the complicated course of treatment our patient was assigned to undergo allogeneic stem cell transplantation in first remission. But, the transplantation was only possible with wounds totally closed. It took another six surgical interventions and nearly eight months to finally close all wounds (see Figure 4). After further recovery the patient underwent allogeneic stem cell transplantation as planned. Today, she is doing well and there is no evidence of disease.

## Discussion

Necrotizing fasciitis is a rare, but one of the most severe and potentially highly lethal soft-tissue infection. If surviving this medical and surgical emergency, patients face long hospitalizations on intensive care units, multiple reconstructive interventions and intensive rehabilitation. Mortality rates have been shown to be determined by the time and delay of operative intervention [6-9]. Therefore, timing and adequacy of radical, extended operative debridement coupled with supportive broad-spectrum antibiotics has a substantial influence on the remaining high mortality [10,11]. To assure patient safety not only in surgery - a high index of suspicion leading to the expeditious diagnosis of necrotizing fasciitis followed by appropriate resuscitation and eradicative surgery is needed. The diagnosis and distinction of NF from other less fatal soft tissue infections is still notoriously difficult and becomes even more challenging if patients’ clinical presentation is exceptional due to an underlying disease and its specific therapy.

There are less than 10 cases reported with adults suffering from necrotizing fasciitis secondary to acute myeloid leukemia (see [12]). The few instances describing such cases underline the necessity to consider NF even if the clinical symptoms, skin manifestation and course of the patient’s condition might not be typical for NF. That is particularly applying for cases of malignancy and following chemotherapy as the subsequent granulocytopenia might mask typical findings like fever and high levels of inflammatory markers in the laboratory diagnostic or lead to missing bacteria and inflammatory cells in the histopathological testing.

We present a young woman with first AML manifestation and subsequent immune suppression due to intensive chemotherapy that went on suffering from a severe form of necrotizing fasciitis of the lower extremity. Our case presents a diagnostic challenge given the equivocal clinical signs – including the skin manifestations and a potentially misleading histology. This report may help increase awareness of untypical presentations of necrotizing soft-tissue infection in severely immunocompromised patients.

Although there have been several attempts to identify special criteria for early diagnosis, necrotizing fasciitis is a clinical diagnosis depending on verifying operative findings. As the infection takes its origin in the deep tissue, delayed diagnosis followed by subsequent delayed operative therapy is still a problem [6,10]. NF often follows bagatelle injury like stiches, injections, trauma or more seldom operation or burn injury. Following such a bagatelle injury, different bacteria are able to induce necrotizing fasciitis like streptococci, staphylococci, enterococci, escherichia coli, and anaerobe bacteria [13]. Characteristically, type II NF is a monomicrobial infection with streptococci or gram-negative bacteria, which shows a more rapid and aggressive clinical course than type I infections. In general, predisposing risk factors are diabetes mellitus, immune suppression, end-stage renal failure, pulmonary disease, malignancy (especially leukemia), and injection drug abuse, which are normally associated with type I infection, whereas immune competent, healthy people are more often confronted with aggressive forms of type II infections [13]. Regarding this case, our patient suffers from a malignancy (AML) and a subsequent immune suppression due to chemotherapy, which normally would have predestinated her to develop a polymicrobial type I infection. But, in our case, we found a monomicrobial type II infection as the taken skin rashes only showed E. coli. Furthermore, E. coli is not a typical type II category bacterium. Most commonly type II infections are induced by group A streptococcal bacteria.

The variety and in some cases even absence of early and specific cutaneous and systemic manifestations may lead to a potentially fatal failure of immediate recognition and diagnosis of necrotizing fasciitis. The commonly described clinical signs are disproportional pain, erythema, tenderness, warm and swollen skin, blistering, skin crepitus and necrosis. But, they are only observed in 10% to 40% of all patients [8,14,15]. All these aforementioned signs are indicative for a local infection, but the differentiation between non-necrotizing soft-tissue infections like cellulitis or erysipelas and necrotizing soft-tissue infections remains quite difficult. Apparently, the most constant initial clinical feature (91,1%) is “pain out of proportion” or so-called “pain extending past margin of apparent skin infection” compared to physical finding [13]. In this presented case, our patient showed only unspecific signs of systemic infection with neutropenic fever and a raise of CRP without any infectional focus two days after last induction chemotherapy (8 days after admission). She did not present any cutaneous signs in the first few days. In the course of two more days she developed a hematoma on the lateral thigh and asking her she remembered a blunt trauma to her lower extremity a few days before. She only reported little pain over the whole period. Signs of severe systemic manifestation like hypotension, prostration or even multiple organ failure have often been described to be helpful in diagnosing patients suffering from NF [16,17], but have not been seen in our case. In accordance, *Wong and colleagues* showed in their retrospective study, that only 18% were hypotensive and only 53% presented with high temperature on admission [8]. Retrospectively, the presented hematoma was not only due to the bagatelle injury but the initial sign of a necrotizing fasciitis. Therefore, one has to keep in mind that patients can appear systemically quite well despite the presence of necrotizing fasciitis especially in case of immune suppression - as these patients are not able to response to infection adequately and skin manifestation may present different due to their blunted immunological response system.

In the last decades, a variety of diagnostic tools have been described to facilitate and hasten the diagnosis of NF. For instance, *Wang et al.* established three different stages of NF according to skin manifestations [18]. They retrospectively analyzed the cutaneous manifestations of NF in patients who were not surgically treated for at least 8 days. Depending on these observations they introduced a staging system correlating with disease’s progression (early (1), intermediate (2) and late (3) stage). Following this clinical staging system, patients in stage 1 typically present with tenderness to palpation, erythema and swollen and warm skin. Patients in stage 2 show blister and bullae formation as well as skin fluctuance and induration. Patients in stage 3 present with hemorrhagic bullae, skin anesthesia, crepitus and skin necrosis. Our patient first did not show any cutaneous manifestation besides a little hematoma after the inadequate trauma to her upper leg without swelling, erythema or warming. In the course of time it rapidly changed and her lateral thigh suddenly presented with a blistering and a central necrosis with a surrounding erythema. Following *Wang et al.* she did not show the typical first and second stage of NF, but the late third one without precedent “cutaneous warning signals”.

Based on laboratory findings, *Wong et al.* proposed a numerical scoring system to distinguish necrotizing fasciitis from other soft tissue infections, called the LRINEC (Laboratory Risk Indicator for Necrotizing Fasciitis) score. This score is calculated by assuming each of the six potentially predictive factors (CRP, WBC, hemoglobin, sodium, serum creatinine, serum glucose). They are measured upon admission to classify patients into a group of low-, intermediate-, and high-risk patients [19]. In our case, we can retrospectively calculate a LRINEC score of 2, which correlates - according to presented scoring system - with a low risk category and a NF probability of less than 50%. One possible source of error in this scoring system might be the fact that normally an increase of leukocytes - as correlating with the systemic infection - leads to a higher LRINEC score. But this is not the case for severely immunocompromised patients who cannot develop leukocytosis and therefore underscore in this scoring system.

*Wall et al.* established another scoring system using WBC count and serum sodium levels to diagnose NF. With a specificity of 76% and a positive predictive value of 26% it might be of help for the elimination of the diagnosis of a necrotizing soft tissue infection but not for the proof of it [20]. Besides the typical inflammatory markers like CRP, leukocytes and PCT, patients suffering from NF often present with hypocalcaemia due to the fat necrosis, acidosis, hypoalbuminemia or hyperuricaemia [21]. Until now, these mentioned laboratory abnormalities in patients with NF are only nonspecific findings.

In order to immediately distinguish between necrotizing and non-necrotizing soft tissue infections a prospective study was performed to characterized serum cytokine levels and WBC counts of patients presenting with a high suspicion of NF in the emergency department. In their study, *Rodriguez et al.* found significantly higher levels of WBC and significantly lower levels of interleukin-1β in patients with NF compared to those suffering from non-NF soft tissue infections. There were no significant differences regarding other potential inflammatory cytokines like Il-1Ra, IL-6, IL-8 or INF-γ [21]. Although these findings might be helpful in the early diagnosis of NF, cytokine assays needed for an instant screening are not widely available, which is - at that time - the major limitation.

*Trebesius et al.* tested fluorescence in situ hybridization (FISH) and showed that it was a suitable method for the rapid and cultivation-independent identification of bacterial pathogens. The results of their experimental study described an rRNA-targeted oligonucleotide set covering more than 95% of the pathogens that are associated with NF. However, they only studied a small sample size and this method is only an experimental approach so far [22].

Particularly for infectious diseases following a fulminant live-threatening course, such as NF, a fast and reliable detection technique would be of great importance. But, taking all these investigations into account, a reliable and widely available test for the contemporary diagnosis of NF is unfortunately still missing.

A further obligatory diagnostic tool, especially in unusual cases in which the intraoperative findings are unclear, is histology. In untypical cases, the result of the tissue sample and instantaneous sections might be very helpful in distinguishing between non-necrotizing soft tissue infection or necrotizing forms and might thereby help the surgeon to decide, whether there is a need for an early second look and repeated debridement. Common pathologic criteria as described by *Stamenkovic and colleagues* to reliably identify NF imply extensive tissue destruction, necrosis of the superficial fascia, dermis-, and fascia-infiltration of polymorphonuclear leucocytes, fibrinous thrombi of arteries and veins coursing through the fascia, angiitis with fibrinoid necrosis of arterial and venous walls, presence of microorganisms within the destroyed fascia and dermis and an absence of muscle involvement [23]. The histopathological findings in our presented case were unusual (see Figure 3). The initial pathological report said that - as there were *no signs of bacteria or inflammatory cells* –a necrotizing fasciitis was more or less improbable. In contrast, our intraoperative findings were typical including the so-called “dishwater” or “foul-smelling discharge”, necrosis and loss of the normal resistance of the fascia to finger dissection, which is called the positive “finger test” [10]. Therefore, our patient was treated according to NF guidelines irrespective of the initial histopathological findings. Regarding our special case, the initial skin biopsy and microbiological testing was misleading. In our opinion, this is most likely due to the fact that our patient suffered from severe neutropenia and thereby could not respond to this necrotizing infection adequately. After beginning recovery, further taken tissue samples then showed the expected typical inflammatory cells and microbiological testing confirmed ESBL infection. Considering these facts - in terms of doubt - the operative exploration should remain the gold standard modality for the diagnosis of NF irrespective of all available diagnostic tools.

The severe granulocytopenia was a key component in our case. Bone marrow suppression - particularly granulocytopenia - is a common feature of AML itself and is an inevitable side effect of intensive treatment of leukemia. Our patient showed a severe neutropenia from right at presentation of disease. Because of persistent neutropenia with evidence of severe necrotizing soft tissue infection, we decided to start G-CSF therapy on day 18. The decision to start a supportive treatment with granulocyte colony stimulating factor (G-CSF) in AML patients is discussed controversially but depends on the circumstances. One the one hand, G-CSF therapy yields a certain risk of leukemic blasts induction. One the other hand, however, administration of this cytokine to neutropenic leukemia patients has been associated with a reduction in fever, clinical infections, and length of hospital stay [24,25]. According to current opinion, leukocyte response correlates well with a favorable outcome in children with NF and leukemia notifying that leukocyte production by bone marrow is essential for survival of leukemia patients with additional necrotizing fasciitis [26-28].

In summary, it is essential to accentuate that there are no mandatory “hard signs” for the diagnosis of necrotizing fasciitis, which still leads to a missed early diagnosis in 85% to 100% of all cases [8]. A retrospective analysis pointed out, that 35% of later NF has been misinterpreted like cellulites or severe non-necrotizing soft-tissue infection [29]. Another retrospective study found that only 14% of patients with NF were admitted with the right diagnosis [8].

For this reason - even more in case of malignancy and immune suppression that might lead to an untypical clinical presentation - patients depend on a high awareness of the involved physicians and a close cooperation of all medical faculties to assure patient safety.

## Conclusion

The combination of malignancy like AML and subsequent severe neutropenia due to chemotherapy increases the risk of delayed diagnosis and consequent surgical treatment of necrotizing fasciitis as “typical” skin signs, systemic manifestations and histopathological findings might be misleading or even missing according to the underlying disease. Delayed recognition of NF leading to consequent massive soft tissue loss and sepsis remains a potential deadly pitfall in the management of this disease. To assure patient safety in surgery, a high amount of suspicion and awareness of the variety of clinical findings is indispensable when confronted with such a complex and fluctuating clinical entity.

## Abbreviations

AML: Acute myeloid leukemia; ESBL: Extended-spectrum beta-lacatmase; FISH: Fluorescence in-situ hybridization; G-CSF: Granulocyte colony-stimulating factor; HAM: High-dose Ara-C and Mitoxantron; HLA-DR: Human leukocyte antigens; Il-1Ra: Interleukin-1 Receptor Antagonist; IL-6: Interleukin-6; IL-8: Interleukin-8; INF-γ: Interferon-gamma; NF: Necrotizing fasciitis; PCT: Procalcitonin; rRNA: Ribosomal ribonucleic acid; WBC: White blood cell.

## Competing interest

The authors declare no competing interest related to this manuscript.

## Authors’ contributions

MB and EE drafted and designed the manuscript. EE, MB and AV performed the clinical examinations and the surgical procedures. AV was the attending oncologist and systematically reviewed this manuscript. KS provided the histopathological pictures and proofed the manuscript for the correctness of the pathological items. VB critically analyzed the literature and finalized the manuscript. WM gave substantial input and critically revised the manuscript. All authors gave final approval of the article to be published.
